# Nanoscale Drug Delivery Systems in Glioblastoma

**DOI:** 10.1186/s11671-022-03668-6

**Published:** 2022-02-16

**Authors:** Zihao Liu, Xiaoshuai Ji, Dong He, Rui Zhang, Qian Liu, Tao Xin

**Affiliations:** 1 Department of Neurosurgery, Shandong Provincial Hospital, Cheeloo College of Medicine, Shandong University, Jinan, 250021 China; 2 Department of Neurosurgery, Shandong Provincial Qianfoshan Hospital, Cheeloo College of Medicine, Shandong University, Jinan, 250014 China; 3 Department of Neurosurgery, Shandong Provincial Qianfoshan Hospital, Shandong First Medical University and Shandong Academy of Medical Sciences, Shandong Medicine and Health Key Laboratory of Neurosurgery, Jinan, 250014 China; 4 Department of Histology and Embryology, School of Basic Medical Sciences, Cheeloo College of Medicine, Shandong University, Jinan, 250012 China; 5 Department of Neurosurgery, Jiangxi Provincial People’s Hospital Affiliated to Nanchang University, Nanchang Jiangxi, 330006 China

**Keywords:** Glioblastoma, Nanoparticles, Biomaterials, Drug delivery systems

## Abstract

Glioblastoma is the most aggressive cerebral tumor in adults. However, the current pharmaceuticals in GBM treatment are mainly restricted to few chemotherapeutic drugs and have limited efficacy. Therefore, various nanoscale biomaterials that possess distinct structure and unique property were constructed as vehicles to precisely deliver molecules with potential therapeutic effect. In this review, nanoparticle drug delivery systems including CNTs, GBNs, C-dots, MOFs, Liposomes, MSNs, GNPs, PMs, Dendrimers and Nanogel were exemplified. The advantages and disadvantages of these nanoparticles in GBM treatment were illustrated.

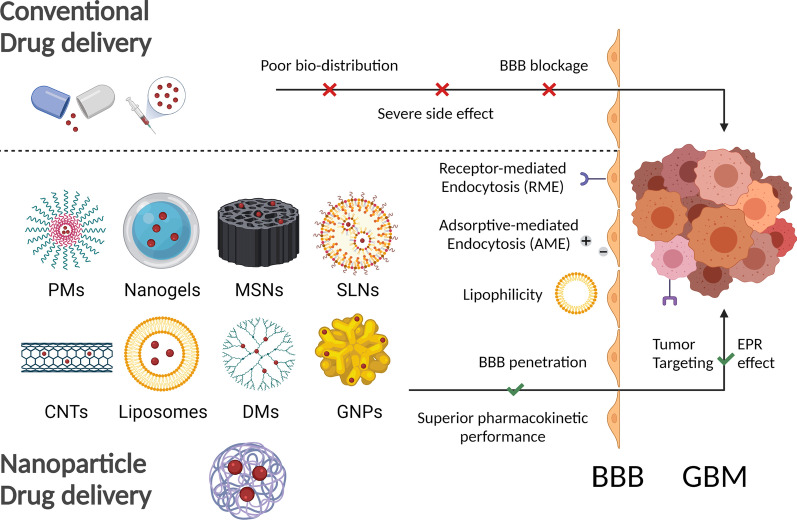

## Introduction

Glioblastoma, also called glioblastoma multiforme (GBM), is considered as the most aggressive cerebral tumor in adults. The standard therapy for GBM is maximal safe resection followed by adjuvant radiation and oral temolozomide (TMZ), which extends patients’ life expectancy only by 16 to 18 months [1]. Due to the aggressive and invasive characteristics of GBM, resecting all tumor tissues in surgery is acknowledged to be impractical. Additionally, extensive resistance can be easily induced after long-term radiotherapy and chemotherapy treatments. The high phenotypic and genotypic heterogeneity of GBM result in multidrug resistance and limited specificity for drug delivery. The bioavailability and efficacies of delivered therapeutic drugs are mainly impaired by factors including tumor microenvironment, stem cell, immune escape, and the most importantly, Blood–Brain Barrier (BBB).

Nanotechnology is an interdisciplinary science to develop and investigate materials at nanometer scale. Disciplines such as physics, chemistry, engineering and biomedical are involved in, hence nanotechnology has become an emerging field in recent years. Although the sensu stricto description of nanotechnology is defined as the manipulation of matters from 1 to 100 nm (by National Nanotechnology Initiative), researchers prefer to recognize it with the broad range to hundreds of nanometers (in submicron scale), especially in the biomedical field. With the remarkable advancements in nanotechnology, nanomaterials were translated into the biomedical area and numerous nanoparticles (NPs) have been developed as drug delivery systems (DDS) for diagnostic and therapeutic application. Based on their different action modes, nanoscale DDS can be classified as passive targeting or active targeting systems. The passive targeting DDS exploit the signatures of tumoral angiogenesis, in which the new blood vessels have enhanced permeability, and the poor lymphatic drainage can passively cause NPs retention (EPR effect). However, due to the existence of intact Blood–Brain Barrier, passive targeting DDS that lack selectivity are unable to penetrate into brain effectively and are unsuited to the GBM treatment. Therefore, active targeting DDS modified with vectors including peptide, protein, aptamer, small molecule and hybrid membrane are utilized in GBM therapy to improve the effectiveness of delivered drugs [2].

Active targeting nanoscale drug delivery systems are able to overcome the limitations of conventional medication therapy. Blood–Brain Barrier is established between cerebral capillaries and neuroglia cells. The tight junctions between endothelial cells selectively restrict the paracellular diffusion of more than 98% particular small molecules including chemotherapy drugs and other therapeutic molecules [3]. The insufficient drug concentration in GBM local will lead to inferior therapeutic efficacy, yet several strategies for NPs bio-modification were employed to enhance the infiltration ability of drug platform. By utilizing Receptor-mediated Endocytosis (RME) process, drug-encapsulated NPs that conjugated to the paired ligands on brain endothelial cells could obtain significantly enhanced BBB permeability [4, 5]. Cationic-joint [6] and lipophilic [7, 8] NPs could effectively transport drugs into the brain through Adsorptive-mediated Endocytosis (AME) and transcellular lipophilic pathway, respectively. Besides, Carrier-mediated Endocytosis (CME) involving nutrient transporters [9] was utilized for effective delivery of nanoscale DDS. Hybrid cell membrane-coated NPs [10] could cross the intact BBB via Cell-mediated transport. Comparing to conventional drug administration routine, modified NPs delivery have the advantages of superior pharmacokinetic performance and high specificity. Prolonged half-time, reduced uptake by reticulo-endothelial system and sustained drug release in tumor sites could reduce the delivery dosage and minimize side effects. Smart biomaterials were also employed to achieve specific bio-mechanical application and controlled drug release, in which environmental stimulants such as pH [11], temperature [12] and near-infrared (NIR) light [13] were exploited. Co-delivery of dual/multiple therapeutic molecules by NPs could be utilized to overcome multidrug resistance and the shortcomings of monotherapy. In addition, nanoscale DDS could be applied in surgical implantation [14], transdermal delivery [15], intranasal delivery [16] and ocular delivery [17].Several NPs including Gliadel®, Doxil®, Lupron Depot®, Marqibo® were approved by Food and Drug Administration (FDA) in clinic applications for various diseases.

In this review, we focused on the nanoscale drug delivery systems that have been proposed for the diagnostics and therapeutics of glioblastoma. The synthesis, functionalization and application of NPs were discussed. The advantages and limitation of each nanoparticle were elaborated thoroughly. Finally, the prospect of future GBM nanoscale DDS therapy was discussed.

## Carbon Allotropes

### Carbon Nanotubes

**Fig. 1:**
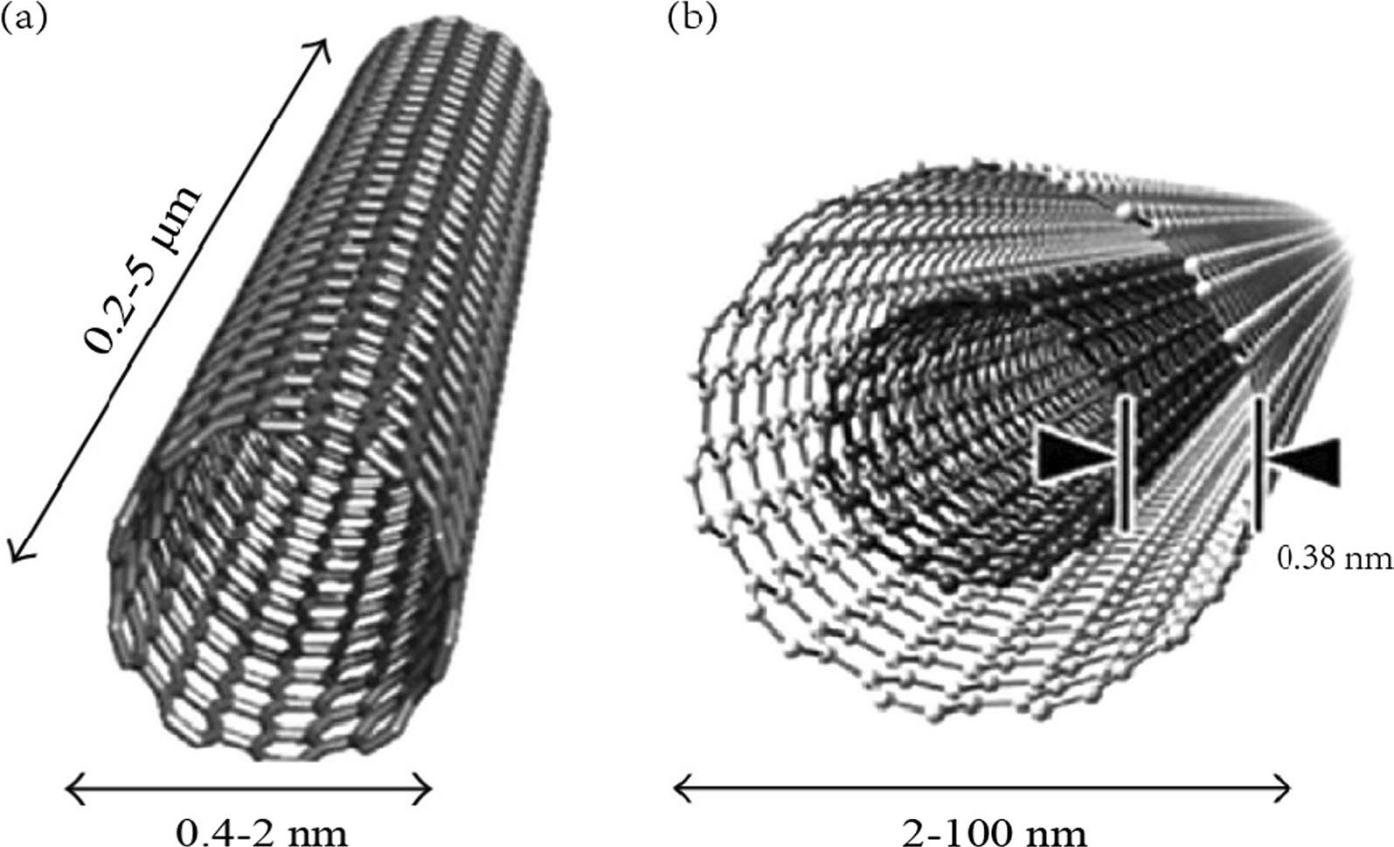
Conceptual diagrams of single-walled carbon nanotubes (SWCNT) (**a**) and multi-walled carbon nanotubes (MWCNT) (**b**). Referred from [23], OA

Carbon nanotubes (CNTs) were recognized as the seamless cylindrical-shape materials with high aspect ratio and satisfactory penetration capacity [18]. CNT has emerged as an influential material in the mechanical and electrical area. They are classified into two structural types: single-walled CNTs (SWCNTs) and multi-walled CNTs (MWCNTs) (Fig. 1). SWCNTs are composed of a single cylindrical graphene sheet with diameter ranging from 0.4 to 2 nm [19]. MWCNTs are multilayer coaxial graphene sheets that encircle an inner cylinder. The outer diameter of MWCNTs ranges from 2 to 100 nm [20]. Although CNTs possess extraordinary properties such as high drug loading efficacy and photoluminescence, limitations including low solubility in aqueous solvent and short half-life impede their applications in life system. Researches [21, 22] indicated that the original CNTs showed inherently toxicity to organism irrespective of the preparation. Functionalization is acknowledged to be an effective approach to improve the dispersion and biocompatibility of CNTs. For instance, by involving hydrophilic groups/polymers, the poor solubility and bio-distribution of CNTs can be significantly improved. The major functionalization of CNTs includes oxidation, acylation, cycloaddition and noncovalent associations, in which the toxicity could be reduced [20]. Consequently, CNTs were exploited as an ideal vehicle for drug delivery in recent years due to the facilely modified features and stability. The advantages and disadvantages of CNTs as drug delivery system were listed in Table 1.

**Table 1:** The advantages and disadvantages of various nanoparticle systems

	Advantages	Disadvantages
Carbon nanotubes	Wide surface area for efficient drug loading High cell permeability Chemical inertness Flexible functionalization	Latent toxicity (Carcinogenicity) Hydrophobicity Immunogenicity Inferior dispersion in body fluid
Graphene-based nanomaterials	Large surface area Acceptable biocompatibility Excellent physical properties Facile functionalization	Hydrophobicity Immunogenicity Potential accumulation
Carbon dots	Simple synthetic materials Diverse synthetic methods Great biocompatibility Photoluminescence	Autofluorescence under UV and damage to adjacent tissues Deficiency of information about the delivery mechanisms
Metal–organic frameworks	Extremely large surface area Easy synthesis and modification Stimuli-responsive system	Relative instability Underlying toxicity Agglomeration Poor dispersion
Liposomes	Excellent biocompatibility Broad adaptability Low immunogenicity Facile fabrication method	Rapid clearance Low stability Low transfection rate
Mesoporous silica	Tailorable mesoporous structure Larger surface area and pore volume Well biocompatibility	Burst release Poor stability Rapid elimination
Gold nanoparticles	Ultra-small sizes Tunable structures Excellent optical properties	Poor elimination rate (retention) Potential toxicity Non-biodegradability
Polymeric micelles	Minimal size Self-assembly pH-sensitive Well biocompatibility	Rapid clearance Off-target effect Dissociation Secondary aggregation
Dendrimers	Small size High molecular uniformity and monodispersity Ease of surface modification Nonimmunogenicity	Nondegradability Cytotoxicity affected by generations and cationic surface
Nanogel	Excellent biocompatibility High stability Large drug loading efficiency Stimulus-responsive capacity Controlled release	Clearance in circulation Uptake by mononuclear phagocytic system

By modified with functionalized groups and polymers on surface, CNTs garnered superior properties such as hydrophilicity, biocompatibility and specificity. Samuel et al. [24] designed chemical functionalized nitrogen-doped MWCNT by acid treatment. The products N-MWCNT-ox specifically eliminated RG2 cells through DNA damage and oxidative stress without inducing reactive inflammation or systemic toxicity. In addition, the combination of N-MWCNT with TMZ exhibited additive tumor-suppressive effects on GBM. Although immunostimulatory oligonucleotide CpG was considered to activate TLR9 as well as initiate immune system to counteract GBM, its efficiency was proved disappointed in vivo. Darya et al. [25] integrated CpG with SWCNT non-covalently, which assisted the delivery of CpG without affecting its therapeutic properties. They demonstrated that SWCNT/CpG inhibited the migration of GBM cells by inducing TLR9/ NF-κB pathway of macrophages. In another efforts, chemotherapeutics including Carmustine [26], Oxaliplatin [27], Lucanthone [28] and Dasatinib [29] were encapsulated into the functionalized CNTs for GBM treatment. Evidences suggested that N-CNTs/Carmustine [26] exhibited continuous kinetic release for 72 h, contributing to the increased intratumoral drug concentration and limited systemic toxicity when comparing with conventional administration route. Besides, TAT-PEI-B-MWCNT-COOH-Oxaliplatin [27] and f-MWNT-ANG [30] showed significantly enhanced BBB penetration and GBM targeting properties in animal model. Therefore, in order to effectively utilize CNTs as drug carriers, the surface modification is an indispensable part.

### Graphene-Based Nanoparticles (GBNs)

**Fig. 2:**
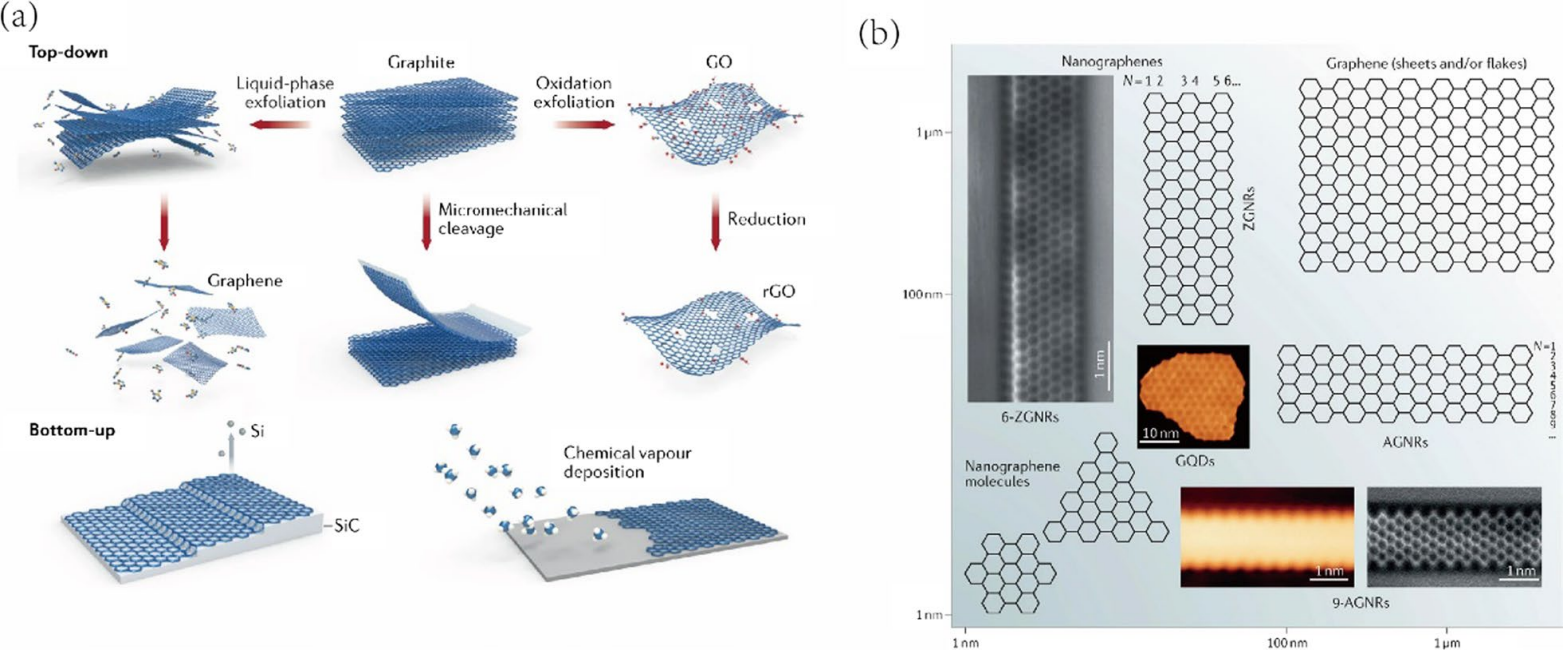
**a** Two main routes to prepare GBNs: “Top-down” splitting approach and “Bottom-up” synthesis approach. **b** Classifications of graphenes based on lateral size. The GQDs represent graphene quantum dots. Referred from [34] with permission

Graphene is recognized as the two-dimensional sp^2^ hybridized carbon material that assembles tightly in a honeycomb-like lattice structure. It has been widely employed in electronic devices (sensor, battery and transistor), aerospace and composite materials. Technically, graphene is recognized as the basic building blocks of other carbon materials such as CNTs and quantum dot [31]. Graphene materials possess excellent optical, electrical as well as mechanical properties and they have emerged as the revolutionary materials in nanoscale processing, biomedicine and drug delivery area. In respect of the physical features, graphene is well known for its exceptional stability, well diathermancy, high mechanical strength and great electrical conductivity. The common derivatives of GBNs are graphene oxides (GO) and reduced graphene oxides (rGO) (Fig. 2). They possess superior biocompatibility and straightforward bio-functionalization capacity [32] when comparing to carbon allotrope CNTs. Additionally, the unique photothermic property of GBNs can be utilized in the stimuli-responsive drug release [33].

The planar structure of GBNs provide large surface for the chemical conjugation of drugs or molecules [35]. Cationic glycoprotein Lactoferrin (Lf) belongs to the transferrin family which can be associated with the Lf receptor overexpressed on BBB endothelia cells. Therefore, the Lf-modified drug delivery systems were commonly utilized to across BBB through receptor-mediated endocytosis [36]. Song et al. [37] decorated GO with Lactoferrin and Fe_3_O_4_ to construct superparamagnetic drug-loaded nanocomposite Lf@GO@Fe_3_O_4_@DOX (Doxorubicin). The NPs performed admirable DOX delivery efficiency as well as exceptional anti-tumor capacity. Similarly, peptide angiopep (ANG)-2 is a specific ligand for the low-density lipoprotein receptor-related protein-1 (LRP-1) expressed on BBB endothelia cells. ANG@GO@DOX designed by Zhao et al. [38] displayed enhanced intracellular uptake and cytotoxicity due to the specific endocytosis capacity. In addition, functionalized GO such as PF127@GO@DOX [39], FA@GO@TMZ [40], PLGA@GO@IUdR [41] and mAb@GO@PPa [42] showed promising therapeutic efficacies, indicating functionalization could significantly improve the BBB transport and GBM specificity.

### Carbon dots (C-dots)

Carbon dots, which is also recognized as carbon quantum dots (CQDs), are composed of dispersed spherical carbon particles (graphite-like core and amorphous oxygen-containing shell) with small size below 10 mm [43]. The synthetic materials of CQDs were readily available, and the synthesis methods of CQDs are multifarious, which are generally based on “top-down” or “bottom-up” approaches [44]. Carbon dots possess unique photoluminescence (PL) properties, hence they are promising nanoparticles for imaging, photocatalysis and photovoltaics applications [45]. In addition, owing to the plentiful functional groups for branching and decorating, C-dots became one of the prominent drug delivery systems in GBM therapy.

The single chemotherapeutic drug administration may easily induce resistance and relapse of GBM after long-term employment. Researches [46, 47] focused on dual drug delivery were conducted to surmount the single drug delivery dilemma, however, the overall particle sizes of NPs are too large to across BBB. Sajini et al. [48] developed a triple conjugated system of C-dots with an average particle size of 3.5 nm, in which transferrin, epirubicin and temozolomide were associated. The triple conjugated system exerted intense cytotoxicity to GBM cells and displayed synergistic effects owing to the dual-drug combination. Wang et al. [49] designed the polymer-coated CODs with DOX and I_6_P_7_ (IL-6 fragment peptide for targeting). Based on the photoluminescence property of special CODs, fluorescence resonance energy transfer (FRET) effect were able to be induced by specific wavelength for DOX release real-time monitoring. In acidic solutions, the drug-encapsulated CODs exhibited increased DOX release similarly with other carbon allotropes. This pH-sensitive releasing performance attributed to the acid-induced protonation of NH_2_ group in DOX and consequent dissociation of π-π interaction [50]. Piumi et al. [51] conjugated Gemcitabine and transferrin protein with CODs for pediatric GBM treatment, in which the outstanding BBB penetration, selective targeting as well as anti-GBM efficacy were observed.

Apart from CNTs, GBNs and CODs, other carbon nanostructures like fullerene and nanodiamond (ND) were also exploited for GBM drug delivery. The DDS based on carbon allotropes including CF@LYS@TEG@MMF [52], ND@DOX [53] (via convection-enhanced delivery) and Polyglycerol@ND@DOX [54] were reported in corresponding anti-GBM investigations. With respect to these carbon allotropic NPs, their biocompatibility and toxicity are greatly depended on the layer number, lateral size, hydrophilicity, and the most importantly, surface chemistry [31]. Hence, proper comprehending of how carbon NPs interact with cells is of significance to improve the biocompatibility and specificity through functionalization,

## Metal–Organic Frameworks (MOFs)

**Fig. 3:**
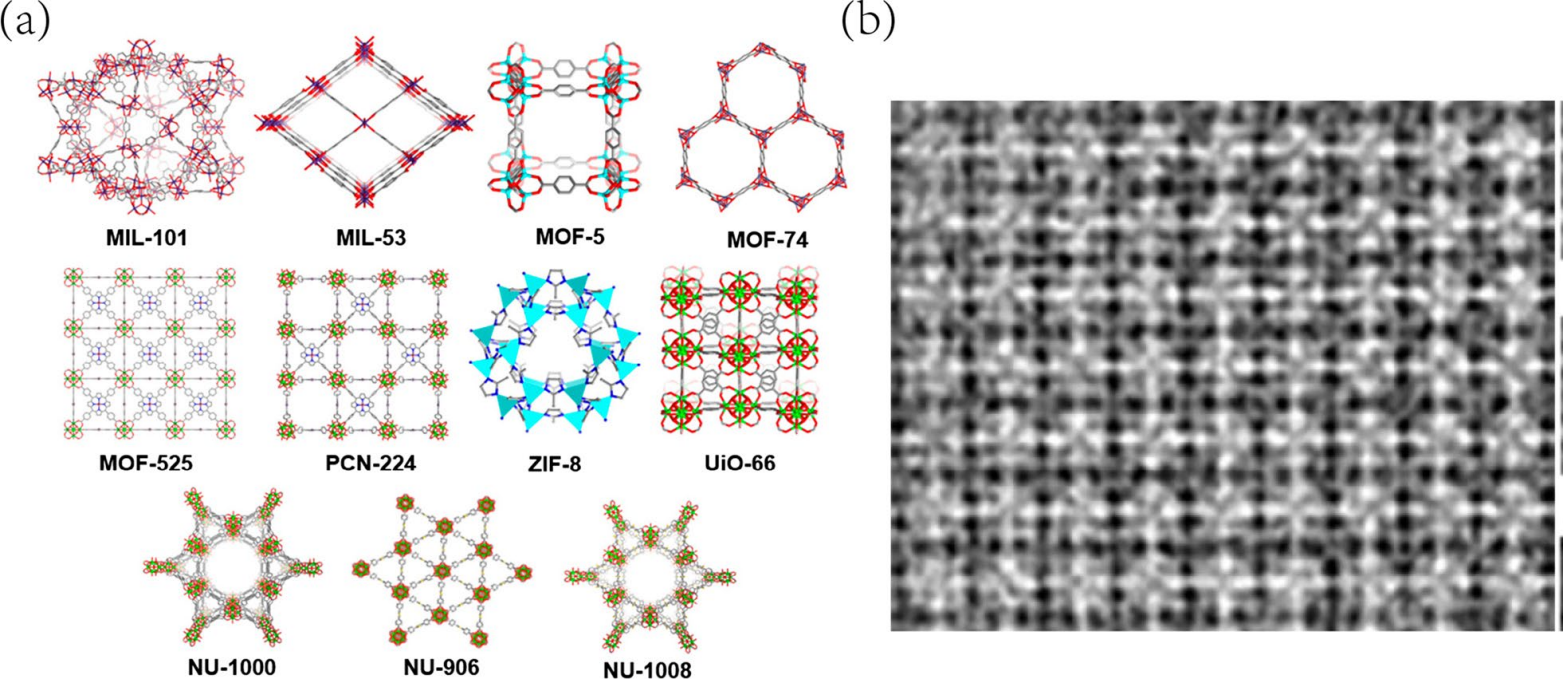
**a** Crystal structures of different MOFs. **b** High resolution TEM image of Uio-66. Referred from [64, 65] with permission

Metal–organic frameworks, the synthetic nanomaterials with mesoporous structure and prominent surface area (1000 ~ 14,000 m^2^/g), are consisted of metal core and coordinated organic ligands [55]. The history of two-dimensional MOFs went back to 1897, when Hofmann [56] fabricated the first hybrid networks of nickel-organic crystal. MOFs were concerned to have pH-sensitive properties and tunable structures (pore size), therefore plenty of them were prepared as ideal drug delivery system. However, the non-biodegradable metal ions including iron, copper, cobalt, cadmium and nickel in MOFs could show potential toxicity to organism [57]. Materials of Institute Lavoisier (MILs) [58], Zeolitic Imidazolate Frameworks (ZIFs) [59], pDBI [60], Hong Kong University of Science and Technology (HKUST-1) [61], University of Oslo (UiOs) [62] and Isoreticular Metal Organic Frameworks (IRMOFs) [63] (Fig. 3) were widely applied in cancer drug delivery, among which ZIF-8 were the most eximious one in GBM investigation.

ZIF-8 is composed of central tetrahedral zinc and coordinated 2-methylimidazole at an angle of 145° [66]. The self-assemble lamellar structure of ZIF-8 was maintained by π–π stacking interaction and hydrogen bonds [66], resulting in the mesoporous cavity and large surface area of ZIF-8. With respect to biocompatibility, zinc represents the second most abundant metal in human, exerting nearly no potential metal cytotoxicity on human cells. Abhijeet et al. [67] developed LND-HA@ZIF-8@Lf@5-FU nanoparticles, in which hyaluronic acid (HA) targeted the CD44 receptors overexpressed on GBM cells, and Lf endowed NPs with BBB penetrating ability. In addition, chemotherapeutics Lenalidomide (LND) and 5-Fluorouracil (5-FU) exerted anti-GBM function in a synergetic manner. Pan et al. [68] produced bimetallic Mn-ZIF-8 aiming to deliver 5-FU. Due to the excessive glycolysis of Warburg effect, pH-sensitive Mn^2+^ and 5-FU were easily released in the relatively acidic GBM microenvironment. Meanwhile, the accumulation of Mn^2+^ in GBM provided available T_1_-weighted MR signals for in vivo imaging, enabling NPs to exert diagnostic and therapeutic effects simultaneously. Zhang et al. [14] constructed modified ZIF-8 system (THINR) wrapped with glioma-associated macrophage membrane (GAMM). Chemokines CXCL10 and GAMM camouflaged with α4β1 integrin provided ZIF-8 NPs with GBM-tropistic property. Chemotherapeutic Mitoxantrone (MIT) and siRNA that targeted endogenous immunosuppressive mediator IDO1 (siIDO1) were co-delivered in ZIF-8 system via intratumor nanogel implantation. The THINR-CXCL10-nanogel restrained relapse of GBM and prolonged overall survival (OS) in tumor-resected animal models.

## Liposomes

**Fig. 4:**
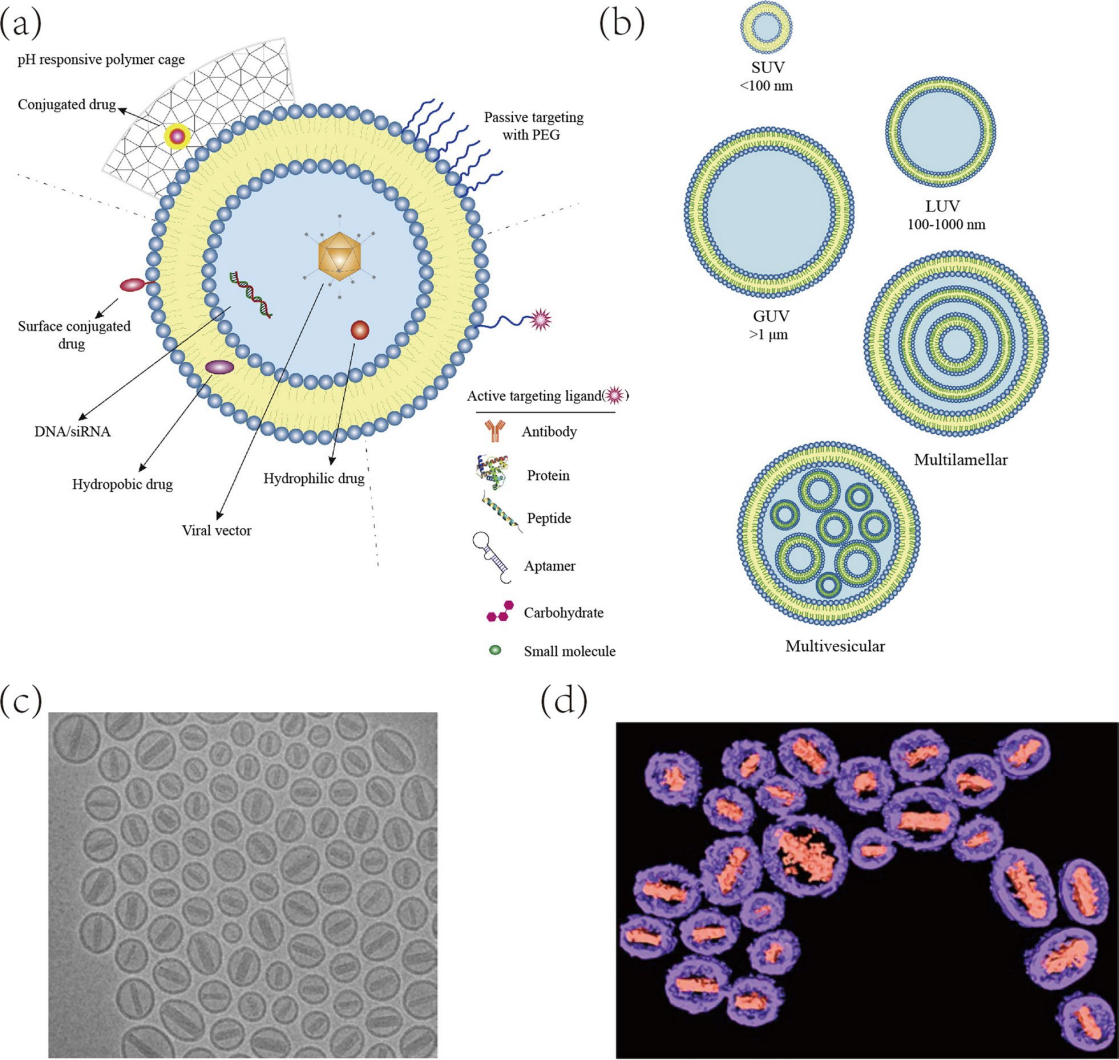
The structure, vesicle size (**a**) and lamellarity classification (**b**) of liposome drug delivery systems. **c**, **d** The cryo-electron tomography liposomes Doxil structure with the liposome density shown in purple and doxorubicin density shown in pink. Referred from [74, 75] with permission

Liposomes are the closed bilayer systems fabricated from self-assembly phospholipids. Depending on the numbers of bilayers as well as method of preparation, the particle size of liposomes ranges from nanometers to micrometers [69] (Fig. 4). Liposomes have attracted substantial attentions in DDS due to their prominent biocompatibility, high loading efficacy, extremely low cytotoxicity and low immunogenicity. The phospholipids are consisted of polar phosphate and hydrophobic lipid tails, which endows liposomes with the ability to encapsulate drug regardless of its physiochemical properties [70]. Drugs can be incorporated into the lipid bilayers [71], sequestered in the hydrophilic core [72] or conjugated on the surface of liposomes [73]. Despite that, the instability and rapid clearance of liposomes in circulation lead to attenuated efficiency, hence invading reticulo-endothelial system is essential for liposomes DDS.

Wei et al. [76] investigated the efficacies of functionalized liposome that loaded with DOX. They synthesized stable peptide ^D^CDX and c(RGDyK) against enzymatic microenvironments through cyclization, partial D-amino acid substitution and retro-inverso isomerization while the GBM-targeting and BBB penetration function remained. Polyethylene glycol (PEG) moiety was adopted as sheath, contributing to the camouflage and prolonged circulation. Butylidenephthalide (BP) was considered to be a promising anti-GBM compound while limited by its hydrophobicity and low bioavailability [77]. Thus, Lin et al. [78] prepared BP-embraced LP@cyclodextrin (a truncated cone shape drug delivery system) complex for expanded loading efficacy and controlled release. In order to evade BBB, the liposomal formulations were administrated through intranasal path. Taken together, the uniqueness of liposomes structure that allows intercalating all kinds of therapeutic molecules highlighted their use in drug delivery.

Apart from liposomes, analogous nanoparticles such as extracellular vesicles (EV), niosomes, solid lipid nanoparticles (SLP), exosomes and microparticles were also acknowledged to be promising vehicles for drug delivery. See in Table 2.

**Table 2:** Liposome particle (LP) based anti-GBM drug delivery system

Drug	Composite	Modification	Detail	Result
Doxorubicin (DOX) [79]	DOX@CB5005@LP	Peptide CB5005 was employed as the cell-penetrating peptide and NF-κB inhibitor	U87 tumor spheroids were prepared to evaluate penetrating ability Cell livability was determined by MTT assay Xenograft-bearing nude mice (subcutaneous and intracranial models) were utilized to evaluate the anti-tumor efficacy	Augmented cellular uptake, increased tumor spheroid penetration and cytotoxicity Enhanced BBB transport and tumor accumulating Prolonged OS
Paclitaxel (PTX) [80]	Rg3@PTX@LP	Rg3 was a substitute of cholesterol as the building block for drug delivery liposomes. RG3 assisted membrane penetration and possessed synergistic effect with loaded anti-cancer drugs	The cellular uptake and cytotoxicity of liposomes was assessed in C6 cells Tumor spheroids and Transwell model were prepared in vitro to evaluate the penetration ability of liposome system The TAM-modulatory effect of PTX was assessed Intracranial xenograft models were utilized to evaluate anti-tumor efficacy	Improved glioma targeting by Rg3-glucose transporter interaction Enhanced BBB transport and PTX cellular uptake The reverse of immuno-suppressive glioma microenvironment
Rapamycin(RPM) [81]	RPM@MTI-31@^D^VAP@LP	^D^VAP was applied as tumor homing peptide	The cellular uptake and cytotoxic activity of liposome MTI-31 were evaluated in U87 cells in vitro Xenograft-bearing nude mice (intracranial models) were utilized to evaluate the anti-tumor efficacy	Increased cellular uptake and anti-proliferative effect against tumor cells High selectivity and glioma targeting property Improved median survival time
Docetaxel (DTX) [82]	DTX@ RI7217@Muscone@LP	RI7217 had high affinity and selectivity for TfR Muscone was a musk ingredient that inhibited the expression of P-gp and MMP-9	Cell livability was determined by MTT assay U87 tumor spheroids and Transwell model were prepared to evaluate penetrating ability U87-MG glioma implantation in nude mice was employed for imaging and survival monitoring	Enhanced BBB transport and tumor penetration Improved targeting ability and anti-glioma effect in vitro and in vivo
Doxorubicin [83]	DOX@P1NS@TNC @ SPIONs@LP	P1NS was GBM-specific cell-penetrating peptide. The anti-GBM antibody TNC and superparamagnetic iron oxide provided targeted delivery property	MTS assays were used to assess cytotoxicity BBB model was established to study liposome permeability The U87 tumor-specific cellular uptake was evaluated by CLMS In vitro stability of liposome and hemolysis assay were conducted	Enhanced BBB penetration Specific tumor tissue targeting Controlled release of encapsulated drugs Increased anti-tumor efficacy and high bio-tolerance
Paclitaxel [84]	PTX@Ang-2@A15@ surviving siRNA@LP	Angiopep-2 could specifically target LPR on BBB surface. A15 was a RNA aptamer that binds to CD133	CCK8 assay and Annexin V-FITC apoptosis assay were used to evaluate the anti-proliferative activity Xenograft-bearing nude mice (subcutaneous and intracranial models) were utilized to evaluate the anti-tumor efficacy	Induced apoptosis of tumor marginal and internal cells Enhanced BBB transport Synergetic chemo-gene therapeutic effect Inhibition of survivin enhanced PTX activity

## Inorganic Nanoparticles

### Mesoporous Silica

Based on the porous structure and well biocompatibility, mesoporous silica nanoparticles (MSNs) are widely applied in biomedicine and drug delivery field. According to International Union of Pure and Applied Chemistry (IUPAC), MSNs are considered to be the ordered silicon oxide structures with pore size ranging from 2 to 50 nm. MSNs possess satisfactory cell bioavailability and show inappreciable cytotoxicity in low dose (< 50 μg/mL) [85]. The synthesis of MSNs is mainly based on silica precursors and surfactants in a template-directed method, which play an indispensable role in determining the pore size and orientation of MSNs products [86, 87]. In order to obtain GBM-tropistic function, modifications including homing ligand and magnetic molecules were introduced to MSNs. Various magnetic-MSNs were designed and the most common magnetic MSNs were composed of metal core and silica shell in an embedded core–shell manner [88, 89]. Apart from that, sandwich-structured [90], hollow-type [91] and rattle-type [92] magnetic MSNs were developed for multidisciplinary applications [93] while seldom were employed in drug delivery.

Zhu et al. [94] developed angiopep-2 modified and lipid coated mesoporous silica (ANG@LP@MSN) to delivery paclitaxel (PTX). The ang-2 and lipid layer contributed to the brain penetrating capacity and effective surface functionalization respectively. In general, MSNs exhibited burst drug release while lipid coating decreased the trend from 46 to 25% in initial 2 h. The T_1/2_ (half life time) and AUC_blood_ (Area under curve of the blood concentration) were increased to 4.5 and 2.5 times respectively comparing with MSNs without lipid layer. The MSN system exerted anti-tumor efficacy (proliferation and migration) both in vitro and in vivo, inducing the cell cycle arrest of GBM cells. Nanoplatform Fe_3_O_4_@mSiO_2_(DOX)@HSA(Ce6) was constructed by Tang et al. [95]. Due to the employment of metal molecule, magnetic guidance ability was achieved by Fe_3_O_4_ core surrounded with silica shell (mSiO_2_). Consequently, aggregation and retention of the whole delivery system in GBM site were observed during magnetic triggering, exhibiting targeting capacity and long-lasting superiority of magnetic nanoparticles. In addition, the combination of photodynamic therapy and chemotherapy (by chlorin e6 and DOX) displayed synergistic antitumor efficacy against GBM. Additional mesoporous silica nanoparticles for GBM therapy were listed in Table 3.

**Table 3:** Mesoporous silica (MSN) based anti-GBM drug delivery system

Drug	Composite	Modification	Detail	Result
Thymoquinone (TQ) [96]	TQ@CS/WA@MSN	Chitosan and stearic acid (CS) shell could associate with GBM cell. Whey protein and gum Arabic (WA) shell promoted the internalization	MTT assay was conducted for in vitro cytotoxicity assessment Apoptosis and cell cycle analysis were detected through Annexin V-FITC flow cytometry Caspase-3 activity and cytochrome-c quantitative analysis were performed	Controlled release of TQ in acid condition Selective cytotoxicity against GBM cells Caspase-3 activation and G2/M arrest in tumor cells
Temozolomide (TMZ) [97]	TMZ@PDA@NGR@MSN	Polydopamine (PDA) and peptide Asn-Gly-Arg (NGR) were designed to target GBM cells	CCK8 assay and Annexin V-FITC apoptosis assay were used to evaluate the anti-proliferative activity in C6 cells The cellular uptake was examined by inverted fluorescence microscope	Enhanced cellular uptake of NPs system Selective cytotoxicity against GBM cells Induced autophagy and apoptosis in C6 cells
Doxorubicin [98]	DOX@CREKA @MSN	Fibronectin-targeting peptide CREKA enhanced selectivity	Vibration caused by external low-power radiofrequency (RF) field induced the drug release from MNS nanoparticle Orthotopic athymic nude mice were utilized to assess anti-GBM efficacy	Enhanced nanoparticle deposition in brain tumor Satisfactory anti-GBM efficacy in vivo
Temozolomide [99]	TMZ@ R8-PNA@MSN	R8 peptide nucleic acids-octaarginine could inhibit miR221	Cell viability assay and apoptosis analysis were conducted FACS and Fluorescence confocal microscopy were utilized to assess cellular uptake	Enhanced cellular uptake and anti-miR211 activity Increased apoptosis of T98 cells in vitro
BSeC [100]	BSeC@cRGD@MSN	αVβ3-targeting cRGD peptide could interact with the endothelial cells on BBB and GBM cells	MTT assay was used to detect cell viability Cell cycle distribution was assessed by flow cytometry U87 spheroids and SD mice were utilized to evaluate the inhibitory effect	Enhanced BBB and spheroids penetration Selective cellular uptake and anti-tumor activity in vitro/vivo Activation of p53, AKT, MAPKs pathways
Arsenic trioxide (ATO) [101]	ATO@ANG@PAA@MSN	Angiopep-2 could specifically target LPR on BBB surface Polyacrylic acid (PAA) was grafted for pH-sensitive release and supporting the lipid membrane	Cellular uptake and intracellular disposition were measured by flow cytometry and LSCM MTT assay was used to evaluate cytotoxicity HBMEC cells were seeded to assess BBB penetration In vivo bio-distribution and anti-tumor study were conducted in SD mice models	pH-responsive and sustained release of ATO Increased BBB transport, enhanced cytotoxicity and inhibition of G2-M transition Satisfactory in vivo bio-distribution and anti-tumor efficacy

### Gold Nanoparticles (GNPs)

**Fig. 5:**
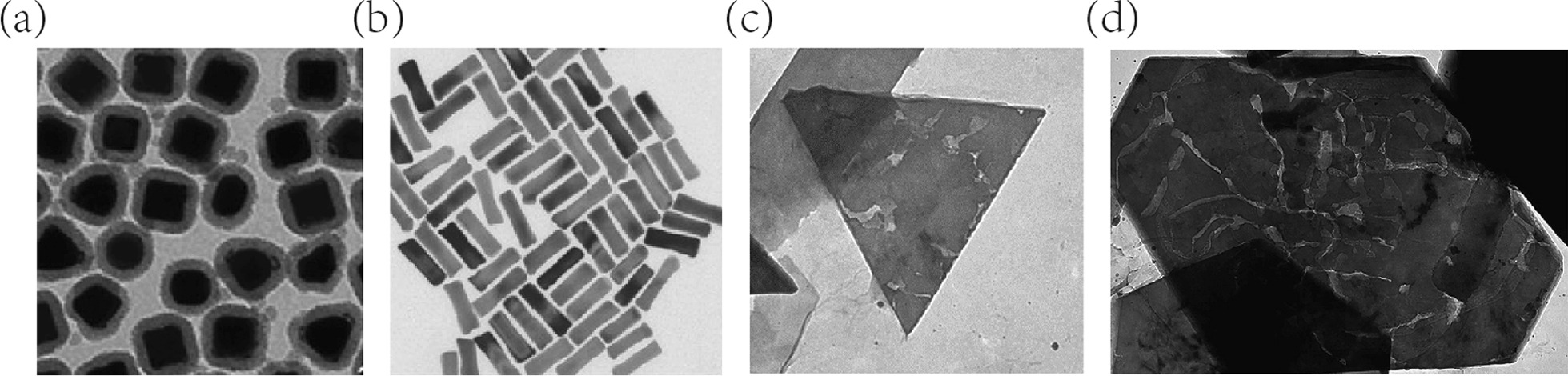
The TEM images of gold nanoparticles with cage-like (**a**), cylindrical (**b**), triangular (**c**) and hexagonal (**d**) morphologies. Referred from [112–114] with permission

Gold nanoparticles, also recognized as AuNPs or colloidal gold, have attracted tremendous attentions on the diagnosis and drug delivery applications of GBM. GNPs have been recommended for antimicrobial applications and showed low toxicity to [102]. Synthetic GNPs have distinct conformations such as spherical [103], cylindrical [104], cage-like [105] and hollow [106] shapes with core sizes ranging from 1 to 150 nm [107] (Fig. 5). Tunable conformations and sizes endow GNPs with unique optical and electrical properties. Surface plasmon resonance (SPR) is the optical phenomenon when GNPs form dipole oscillation in response of incoming light [108]. It has been explored in local induction heating, imaging, biosensor analysis and drug release [107]. The synthesis of GNPs is mainly based on colloidal synthesis method, in which the metal precursor, reductant and stabilizer were applied sequentially [109]. It was elaborated that the size of GNPs profoundly affected the bio-distribution in circulation. Investigation [110] revealed that GNPs with different sizes (10, 50, 100, 250 nm) exhibited distinct distribution patterns after intravenous administration. The depositions of gold were detected through inductively coupled plasma mass spectrometry (ICP-MS) and only the 10 nm GNPs were found accumulated in organs such as testis, thymus, heart and brain. The functionalizations on GNPs including peptides, antibodies and drugs, which are feasible due to the negative charge on surface [111].

Researchers [115] investigated the efficacies of functionalized GNPs that were co-loaded with DOX and HCQ (hydroxychloroquine). Programmed death 1 (PD-1) is a critical immunosuppressive receptor located on T cells, and the overexpression of which indicates the T cell depletions and immune escape in various diseases including GBM. Evidence [116] suggested that impeding PD-1/PD-L1 pathway contributed to the potential antitumor immunity. PD-L1 antibody was associated with GNP drug delivery system [115], which was consisted of peptide (Ala-Ala-Asn-Cys-Lys) conjugated polyethylene glycol-thiol (PEG-SH) and 2-cyano-6-amino-benzothiazole (CABT) conjugated PEG-SH. The modifications of peptide and CABT on GNPs significantly reduced the protein corona effects caused by intravenous administration. The stable physicochemical properties of GNPs contributed to the controlled release of DOX and HCQ in GBM local. In addition, HCQ was reported to resensitize GBM by inhibiting DOX-induced autophagy, which exerted excellent anti-GBM capacity in combine with PD-1/PD-L1 pathway blocking. Gallic acid (3,4,5‐trihydroxybenzoic acid, GA) is a well‐established antioxidant that shows potential anti-tumor capacity against GBM in vitro, however, the limited delivery method and poor drug availability restrict its further application. Through physical agitation adsorption, Zhou et al. [117] constructed the GA@GNPs characterized with an average diameter of 23 ± 0.34 nm. The GA@GNPs system exhibited cytotoxic effect on GBM cells and sensitized the radiation‐mediated S and G2/M cell cycle arrest.

Inorganic nanoparticles such as calcium phosphate nanoparticles (CPNs) [118], layered double hydroxides (LDHs) [119] and halloysite clay nanotubes (HNTs) [120] showed therapeutic benefits in pre-clinical GBM settings. In addition, other metal nanoparticles were designed for GBM therapy such as sliver nanoparticles (AgNPs, especially for enhancing radiosensitivity), copper oxide nanoparticles (CuONPs), zinc oxide nanoparticles (ZnONPs), iron oxide nanoparticles (IONPs), gadolinium-based nanoparticles (GdNPs, for enhancing radiosensitization) and manganese oxide nanoparticles (MnO_2_NPs). The metal NPs based drug delivery systems were listed in Table 4.

**Table 4:** Metal nanoparticle based anti-GBM drug delivery system

Drug	Composite	Modification	Detail	Result
Verapamil (VRP) [121]	VRP@BSA@AS1411@AgNP	Aptamer AS1411 could specifically bind to nucleolin on GBM cells and inhibit P-glycoprotein (P-gp) efflux activity	The cytotoxicity was evaluated by MTT assay in U251 cells Colony formation assay and tumor-bearing nude mice model were employed to examine the radiosensitizing potential	Increased accumulation and cytotoxicity in vitro/vivo Radiosensitizing effect of AgNP composite Decreased TrxR activity
Cisplatin (Pt) [122]	Pt@si-GPX4@FA@IONP	Folate acid was applied as tumor targeting ligand	Cell viability and proliferation were assessed using the CCK-8 assay Lipid peroxidation levels were detected by MDA assay Superoxide anion levels were detected using DHE assay	Increased apoptosis and ferroptosis in U87MG cells Selective uptake of IONP Outstanding therapeutic effect in nude mice models
Temozolomide [123]	TMZ@miR-100@antimiR-21@PEG-T7@GION	GBM cell-targeting T7 peptide was coated on GION shell	Cellular uptake was estimated by fluorescence microscope and flow cytometry Cell viability and apoptosis were assessed by PI and TUNEL staining The GION system was administrated through intranasal method in nude mice model	Sensitized GBM cell to TMZ therapy Upregulated p53, PTEN, PDCD4 and Caspase-3 levels Satisfactory therapeutic outcome in vitro/vivo
HAPtS [124]	HAPtS@CPTES@SPION	HAPtS was a synthetic derivative of Trans-resveratrol, acting as antioxidant	Oxygen radical absorbance capacity (ORAC) assay was conducted as antioxidant test Clonogenic survival, FDA and MTT assay were utilized to assess cytotoxicity	Enhanced cytotoxicity against C6 cells in vitro Damage on plasma membrane instead of mitochondrial metabolism
Paclitaxel [125]	PTX@LinTT1@IONW	Tumor homing peptide LinTT1 peptide was a recently identified ligand of p32	Cellular uptake was evaluated by flow cytometry The in vivo bio-distribution and therapeutic effect were assessed in nude mice model	Accumulation of IONW systems in tumor site Inhibited tumor growth in vivo

## Polymeric Micelles (PMs)

**Fig. 6:**
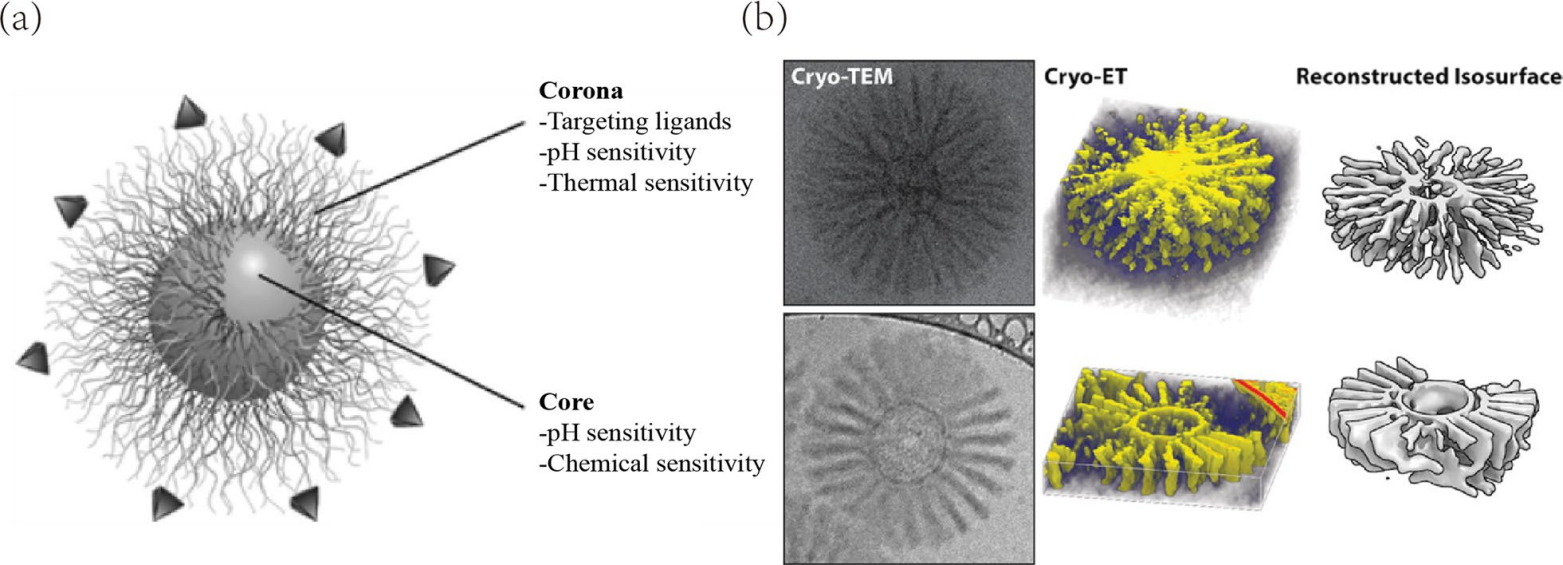
**a** Schematic illustration of the core–shell structure of a polymer micelle. **b** Cryogenic transmission electron microscopy (cryo-TEM), tomography (cryo-ET) and computational 3D reconstruction of multicompartment micelles. Referred from [135, 136] respectively with permission

Polymeric micelles are the sphere-like colloidal particles consistently ranging from 10 to 100 nm [126]. PMs are composed of self-assembly copolymers, which typically possess the analogous conformations similar to phospholipids and surfactants: a hydrophilic domain and a hydrophobic domain. Apart from the most common amphiphilic copolymers (di-block) micelles, PMs that consist of tri-block copolymers (hydrophilic-hydrophobic-hydrophilic) [127, 128] and graft copolymers [129, 130] were developed for the availability of additional functionalization. Comparing to other types of DDS, PMs could act as solubilizing agents for the replacement of toxic solvents [131]. PMs with different morphologies (spherical [132], cylindrical [133], lamellar [134]) garner distinctive biological and pharmacokinetic properties, among which the spherical assembly micelles were extensively elaborated (Fig. 6).

The inward-facing hydrophobic segments of copolymers serve as core in spherical PMs. In order to associate with the interior drugs, hydrophobic segments that possess various functional groups are commonly constructed by polyesters such as PLA [137], PGA [138] and PCL [139]. In addition, the camouflage outer layers of PMs play a pivotal role in stability and targeting. Numerous evidences [140, 141] have indicated the particular functions of polyethylene glycol (PEG) on drug sustained release and surface modification. The external modification of biomaterials with PEG could reduce the immunogenicity of NPs, provide grafting sites and improve the surface absorption.

The micellization of amphiphilic copolymers in aqueous media is mainly dependent on hydrophobic interactions (attractive force) and occurs automatically when concentration reaches the critical micelle concentration (CMC). The CMC of each individual PMs (ranging from 10^–7^ to 10^–6^ M) is mainly affected by environmental temperature, copolymer structure and relative molecular weight [142]. Copolymers start to disassociate when concentration remains below CMC, which results in off-target effects and rapid clearance of formulation by circulation. Dispersity (D) or polydispersity index (PDI), is measured as a crucial parameter for micellization performance assessment. Evidence [73] suggested that copolymers with low dispersity (D < 1.2) were suitable for controlled drug delivery system. Therefore, determining CMC and PDI are indispensable for the development and application of PMs.

Owing to the minimal size and unique micellar structure, PMs delivery system plays an essential role in smart releasing and local accumulation comparing to conventional drug administration method. Typically, water-insoluble drugs were encapsulated in PMs to gain augmented loading capacity. It was acknowledged that cyclic peptide Arg-Gly-Asp (cRGD) could specifically bind to the αvβ3 and αvβ5 integrins overexpressed on GBM cells. The cRGD installed epirubicin (EPI)-loaded polymeric (Acetal-PEG-b-PBLA) micelles were constructed by Quader et al. [143] for GBM treatment. Specifically, the hydrazide functional groups were involved in copolymers and the constituent hydrazine-bond would contribute to the pH-sensitive property. siRNA has been recognized as promising therapeutic agent to induce target gene silencing, however, it was limited by instability and low bioavailability in vivo. Peng et al. [144] focused on the cationic polymers Poly(ethylenimine) (PEI) which was reported to facilitate lysosomal escape of siRNA through proton sponge effect [145]. Tri-block polymeric micelle TMZ-FaPEC (Fa-PEG-PEI-PCL)@siRNA were designed to silence antiapoptotic BCL-2 gene, in which the folic acid (FA) moiety could specifically bind to the folate receptors on GBM cells. Consequently, the intracranial injection of micelle DDS exhibited significant GBM inhibition and survival benefit. The additional polymeric micelles for GBM therapeutic drug delivery employments were listed in Table 5.

**Table 5:** Polymeric micelle (PM) based anti-GBM drug delivery system

Drug	Composite	Modification	Detail	Result
Camptothecin (CPT) [146]	CPT@PEG@iRGD@IR780	Internalizing RGD peptide possessed tumor-targeting motif CendR	Photosensitizer IR780 was loaded for combination therapy and NIR laser acted as a light-triggered switch Cytotoxicity and penetration capacity were determined in U87 cells and spheroid Anti-tumor effect was evaluated in nude mice model	Controlled drug release and augmented ROS generation after laser irradiation Enhanced cellular uptake, BBB penetration and cytotoxicity Excellent anti-GBM efficacy in tumor-bearing mice
Paclitaxel [147]	PTX@L-VAP/RI-VAP@PEG-PLA	l-VAP (SNTRVAP) is a tumor homing peptide exhibiting high binding affinity in vitro to GRP78 protein overexpressed on tumor cells	The cellular, tumor spheroid, in vivo uptake of PM nanoparticle were detected Cytotoxicity was determined by MTT assay Nude mice model was established for in vivo anti-tumor study	Targeting ability for BBTB and U87MG Greatly enhanced drug delivery efficiency and cytotoxicity Augmented in vivo anti-tumor efficacy
Paclitaxel [137]	PTX@TfR-T12@PEG-PLA	TfR-T12 peptide could mediate the drug system to cross the BBB and specifically recognize tumor cells	Flow cytometry and CLSM were utilized to assess cellular uptake (DiR) Cytotoxicity, apoptosis, migration/invasion assay were conducted in U87MG cells Subcutaneous and orthotopic nude mice tumor model was constructed	Enhanced cellular uptake, apoptosis and endocytosis across BBTB Inhibited proliferation, migration and invasion Suppressed tumor growth in vivo
Temozolomide [148]	TMZ@siPLK@ANG2@PEC	Ang2-modified polymers could penetrate the BBB through receptor-mediated transport and accumulate in the brain in large quantities	Flow cytometry and CLSM were utilized to assess cellular uptake (FAM-siRNA) Cell cycle and apoptosis assay were performed through flow cytometry MTT assay was conducted in LN-299, T98G and U87 In vivo anti-tumor efficacy and organ safety was evaluated in nude mice	Enhanced cellular uptake, cytotoxicity and apoptosis in tumor cells G2/M arrest caused by siPLK Enhanced TMZ sensitivity in U87RT No systemic toxicity in tumor-bearing mice
Curcumin (CUR) [149]	CUR@miR21ASO@DP	Deoxycholic acid-conjugated polyethylenimine (DP) was synthesized with deoxycholic acid (DA) and low-molecular weight polyethylenimine	MTT assay, Annexin V/PI assay, and TUNEL assay were performed to evaluate anti-tumor effect in vitro Sprague Dawley rats were used to establish xenograft intracranial glioblastoma model	Induction of PDCD4 and PTEN by intratumorally injected NPs Satisfactory anti-tumor effect in vitro and in vivo

## Dendrimers

**Fig. 7:**
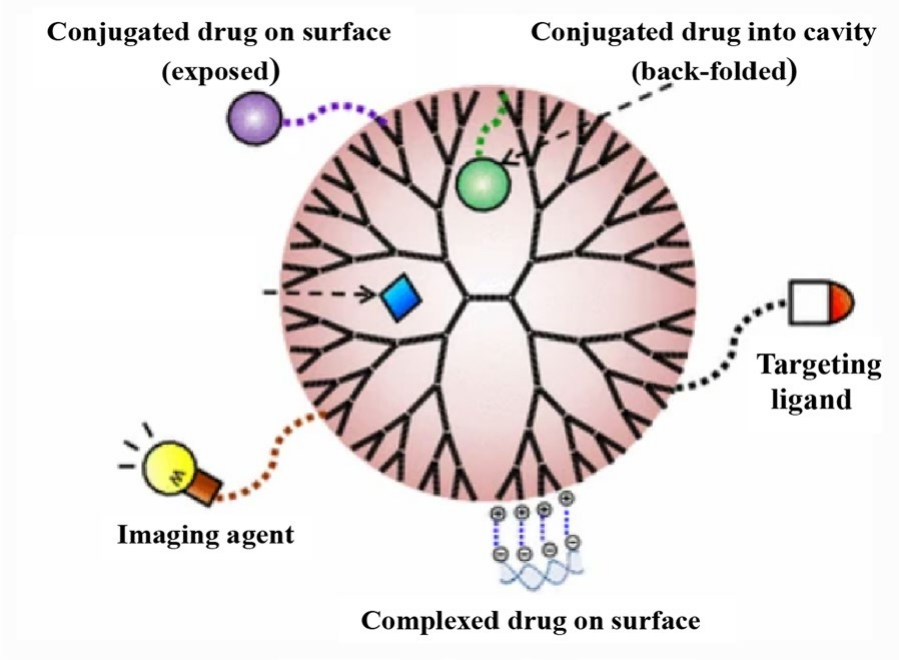
Schematic representation of pharmaceutical applications of dendrimers. Referred from [164] with permission

Since polypropylenimine (PPI) and polyamidoamine (PAMAM) were firstly developed by Buhleier [150] and Donald [151] respectively, the synthetic dendrimers have emerged as profound drug delivery vehicles for their varied applicability. Dendrimers are well known for the dendritic architecture consisting of internal core and repeated external branching units [152]. The layers of repeated polymers are also recognized as generations (G) [153]. It was reported that the increase of one generation led to practically double molecular mass of dendrimers [154]. In general, the toxicity of dendrimers depends on generations, surface group and terminal moieties [155]. Introducing chemical modifications could decrease the cytotoxicity while maintaining advantageous properties [155]. The peripheral branching framework of dendrimers provides adequate graft sites for functional groups and bioactive drugs (Fig. 7), which are typically achieved by covalent conjugation, hydrogen bonding or electrostatic adsorption [156]. Additionally, segmented cavities between polymer blocks allow drug entrapment. Dendrimers can be synthesized through divergent and convergent methods, in which the conformations are constructed in the procedures of core-to-shell and shell-to-core manners respectively [157]. Depending on various compositions and generations, dendrimers and the derivatives possess engineerable sizes ranging from 1 to 15 nm [156]. In recent years. a variety of dendrimers are developed for drug delivery, including PAMAM [158], PPI [159], PLL (Poly-l-lysine) [160], PHH (phosphorus) [161], carbosilane [162] and janus [163] dendrimers. The small sizes and flexible surface modifications provide practicable BBB penetrating and GBM targeting ability for dendrimers.

Tumor-associated macrophages (TAMs) were recognized as an oncogenic factor in GBM. It was demonstrated that the infiltrating and resident macrophages/microglia could be reprogrammed by GBM cells, and the numbers of which were positively correlated with GBM grades [165]. Sharma et al. [166] focused on TAMs and designed PAMAM-OH dendrimer-based rapamycin (Rapa) conjugate. The PAMAM dendrimers with four generations were developed based on ethylenediamine core. Rapa is considered to be a promising anti-GBM reagent towards mTOR pathway and the inhibition of mTOR pathway in TAMs can induce GBM regression. Since Rapa possesses low bioavailability and extremely low aqueous solubility, PAMAM vehicle provided alternative delivery approach across BBB for Rapa administration. The DDS exhibited profound anti-tumor effects by targeting TAMs and GBM cells in vivo. Zhao et al. [167] modified PAMAM with peptide CREKA, which is characterized as tumor-homing ligand towards peritumoral fibrin deposition [168]. The G5 PAMAM was prepared with PEG-CREKA and the average of nanoparticles size was 7.52 ± 0.35 nm. In vivo imaging spectrum (IVIS) results revealed that PAMAM-PEG-CREKA had enhanced retention effect in GBM area, therefore CREKA coated PAMAM was expected to be a potential delivery system for GBM treatment. The additional researches for GBM dendrimers DDS were listed in Table 6.

**Table 6:** Dendrimer based anti-GBM drug delivery system

Drug	Composite	Modification	Detail	Result
Celecoxib (CXB) [169]	CXB@G3BC31	Biotinylated third generation of the poly (amidoamine) dendrimer was substituted with 31 CXB residues	The cellular uptake, proliferation, migration and apoptosis were assessed in U-118 cells Model organism nematode Caenorhabditis elegans was used in toxicological studies	Cellular accumulation of dendrimer NPs in the lysosomes of tumor cells Up-regulated COX-2 level and cell cycle arrest Anti-tumor effect in vitro
Arsenic trioxide (ATO) [170]	ATO@cRGD@mPEG@PAMAM	cRGD can bind to αvβ3 integrin peptides and combine with the integrin receptors on BBB	Cellular uptake, hemolytic toxicity and cytotoxicity were detected in C6 cells Intracellular disposition and BBB penetration were assessed Therapeutic efficacy was evaluated in Wistar rats model	Enhanced BBB penetration and intracellular disposition Cell cycle perturbations in G2/M phase Excellent therapeutic effect in vitro/vivo
TRAIL [171]	TRAIL@Tf@PAMAM	Transferrin (Tf) receptor were overexpressed on BBB and utilized for facilitating brain targeting	Cellular uptake and apoptosis were determined in C6 cells Tumor xenografted SD rat model was established MRI imaging was utilized to monitor therapeutic effect	Accumulated NPs in brain tumor site Induced cell apoptosis Prolonged mean survival time and decreased tumor volume
Rapamycin (RAPA) [172]	RAPA@ PAMAMOH	Hydroxyl PAMAM dendrimers (PAMAMOH) had the unique ability to target activated microglia	BV2 microglia were activated with IFNγ for inflammation analysis Anti-proliferative activity in GL261 cells was detected by MTT assay Systemic toxicity and anti-tumor evaluation were completed in C57BL/6 orthotopic mice model	Ablation and repolarization of TAMs Augmented anti-proliferative and tumor specificity properties in vitro Improved therapeutic efficacy in vivo
Doxorubicin [173]	DOX@Borneol@ANG-2@PAMAM	Angiopep-2 could specifically target LPR on BBB surface Borneol promoted the permeation of drugs across BBB and enhanced their distribution in the brain tissue	Cellular uptake and intracellular disposition were detected through LSCM in HBMEC cells BBB penetration model was established In vitro cytotoxicity on HBMEC and C6 cells were evaluated	Sustained pH-sensitive DOX release Enhanced cytotoxicity and BBB penetration Promoted tumor specific NPs uptake level

## Nanogel

**Fig. 8:**
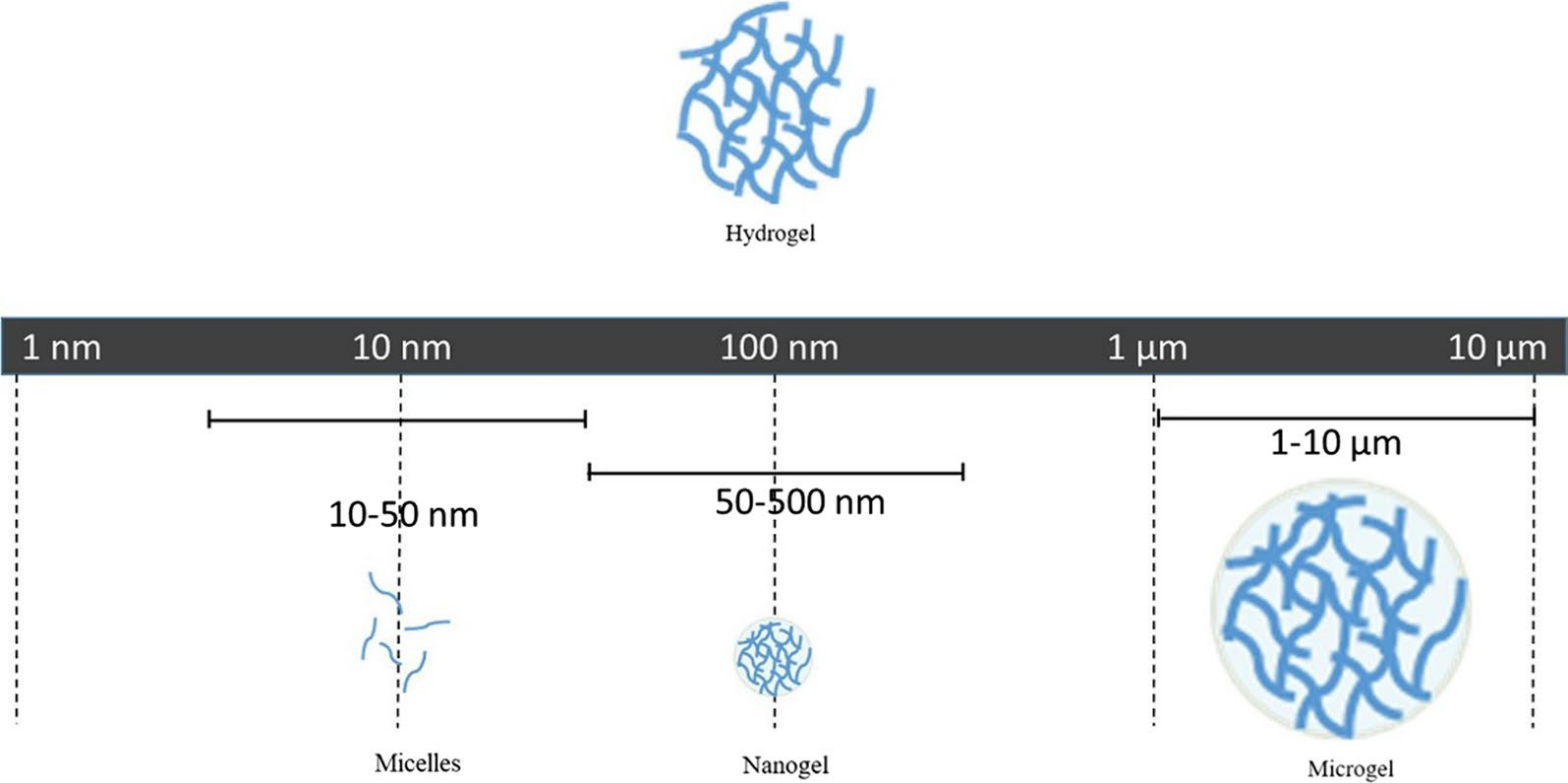
Schematic representation of the network construction of hydrogels, micelles, nanogels and microgels. Referred from [175] with permission

As an emerging class of synthetic and multifunctional polymers, nanogel have garnered immense attentions in tissue engineering and pharmaceutical fields. Nanogel represents the combination of “hydrogel” and “nanoparticles”. It is composed by cross-linked hydrophilic polymers networks and characterized as tunable sizes (50–500 nm) and admirable elasticity [174] (Fig. 8). The construction of nanogel is based on physical crosslinks (van der Waals, electrostatic interactions and hydrogen bonding) and/or chemical covalent bonds, endowing nanogel with the conformational changeability property against environmental stimuli [175]. Nanogel particles can be synthesized in an initiated [176] or self-assembly [177] manner, through which initiator/gelator is required or not respectively. Unlike micelle, the amphiphilic associations between nanogel polymers exhibited superior stability, which is attributed to the plentiful cross-link points in nanogel instead of di-/tri-blocks structure in micelles. Numerous hydrophilic groups (such as -OH、-COOH、-CONH_2_ and -SO_3_H) and porous cavity were possessed by nanogel [178], which contributed to the swelling (water absorption) instead of dissolution of the polymeric conformation in aqueous condition. The imperative consideration on toxicity is of significance for nanogel. Consequently, natural polymers such as alginate [179], amylopectin [180], hyaluronic acid [181] and chitosan [182] have been utilized as building blocks for nanogel drug delivery system.

The release of encapsulated drugs is mainly based on passive diffusion. The swelling of nanogel enlarges the interior mesh size and allows the entrapped drugs release. Therefore, micro-molecules are prone to release in a burst manner while macro-molecules (protein, peptides) in nanogel exhibit sustained release [174]. Additionally, the degradation of physical/chemical bonds between polymeric chains results in hydrolysis of whole delivery system and drug release. In this regard, factors that can trigger the swelling or degradation of polymeric networks enable nanogel to perform stimulus–response property [183]. Environmental factors such as temperature, pH, ionic change, redox and light are exploited as stimuli for the responsive controlled drug release. As described before, the microenvironment of tumor possesses lower pH level due to excessive apoptosis and Warburg effect when comparing to blood and normal tissue (pH 7.4). The methacrylated hyaluronic acid (MAHA) nanogel [184] was designed for pH-sensitive drug release against GBM. Cross-linking gelator MA-OE-MA forms ortho ester linkages with MAHA via aqueous dispersion polymerization. The acid-labile ortho ester bond was effectively sensitive to mildly acid condition [185]. Therefore, the hydrolysis of chemical bonds between polymers and gelators lead to the release of embedded DOX, achieving anti-GBM capacity and tumor targeting. Thermos-responsive nanogel with negative sensitivity is characterized by lower critical solution temperature (LCST). At the temperature lower than LCST, the polymers form hydrogen bonds with water and exhibit in a swollen state. When the temperature reach LCST, the hydrophobic interaction between polymers dominate and the conformation of nanogel appear to shrink and collapse [183, 186]. Lu et al. [187] integrated GO into chitosan nanogel to transport irinotecan, cetuximab and SLP2 shRNA against GBM. The nanogel system exhibited thermo-sensitive characteristic with phase transition temperature/LCST at 32 °C. At room temperature, GO that loaded with drugs and shRNA were embedded into the liquid phase nanogel. The injection of chitosan nanogel into GBM area (> 32 °C) enabled the dehydration and solidification of nanogel, contributing to the subsequent sustained release of anti-tumor agents in situ. The additional nanogel DDS for GBM therapeutic employments were listed in Table 7.

**Table 7:** Nanogel based anti-GBM drug delivery system

Drug	Composite	Modification	Detail	Result
Teriflunomide (TFM) [188]	TFM@ Gellan gum@ Carbopol 974P	Gellan gum and carbopol 974P were applied as gelling and mucoadhesive agents	Ex vivo nasal permeation was performed by using vertical Franz diffusion cell Cellular uptake, bio-distribution and proliferation were evaluated in U87MG cells Systematic toxicity and bio-distribution were determined in Swiss albino mice model	Enhanced nasal permeation and brain accumulation through i.n. delivery High cytotoxicity potential against tumor cells No hepatotoxicity, nephrotoxicity and hematological toxicity
Doxorubicin [189]	DOX@Lf@PBA@HA	Lactoferrin was coated onto the nanogels to achive blood brain barrier penetration Hyaluronic acid and phenylboronic acid provided dual targeting for tumor	Cellular uptake and disposition were determined through flow cytometry and CLMS B.End3/G422 coculture model was used to study the transport ability SD rats and ICR mice were utilized to investigate anti-tumor efficacy and bio-distribution respectively	Triggered drug release manner by high GSH Increased cellular uptake through clathrin and caveolin-mediated endocytosis Optimized bio-distribution and higher permeation ability
TSPO ligand [190]	TSPO ligand-DEX@NG	Cholesterol and porphyrins are known as endogenous ligands for Translocator protein	MTT assay and fluorescence microscopy were utilized in C6 cells Swelling study and rheological study were used to analyze the viscoelastic properties	Proven cellular uptake and cytotoxicity in vivo Excellent stability and controlled release of NG system
TMZ&PTX [191]	TMZ@PTX@PEG-DMA	Intratumoral implantation was used instead modifications on NG	Clonogenic assay was conducted Orthotopic U87MG human GBM tumor resection nude mice model was estabilished	Synergistic effect of PTX and TMZ on U87MG cells Perisurgical drug delivery in the resection cavity
Cisplatin [192]	CPT@Cx43 mAb@BSAT1 mAb@NG	The preferential expression of membrane protein connexin 43 (Cx43) and brain-specific anion transporter (BSAT1) in the tumor was employed for targeted drug delivery	MTS assay was used to evaluate in vitro cytotoxicity on C6 cells Intracranial inoculation of rat glioma 101/8 was constructed Tumor volume was determined by MRI scanning	Higher passive penetration across endothelial barrier Increased drug delivery efficacy and internalization in tumor site Prolonged survival time for animal model

## Discussion

**Fig. 9:**
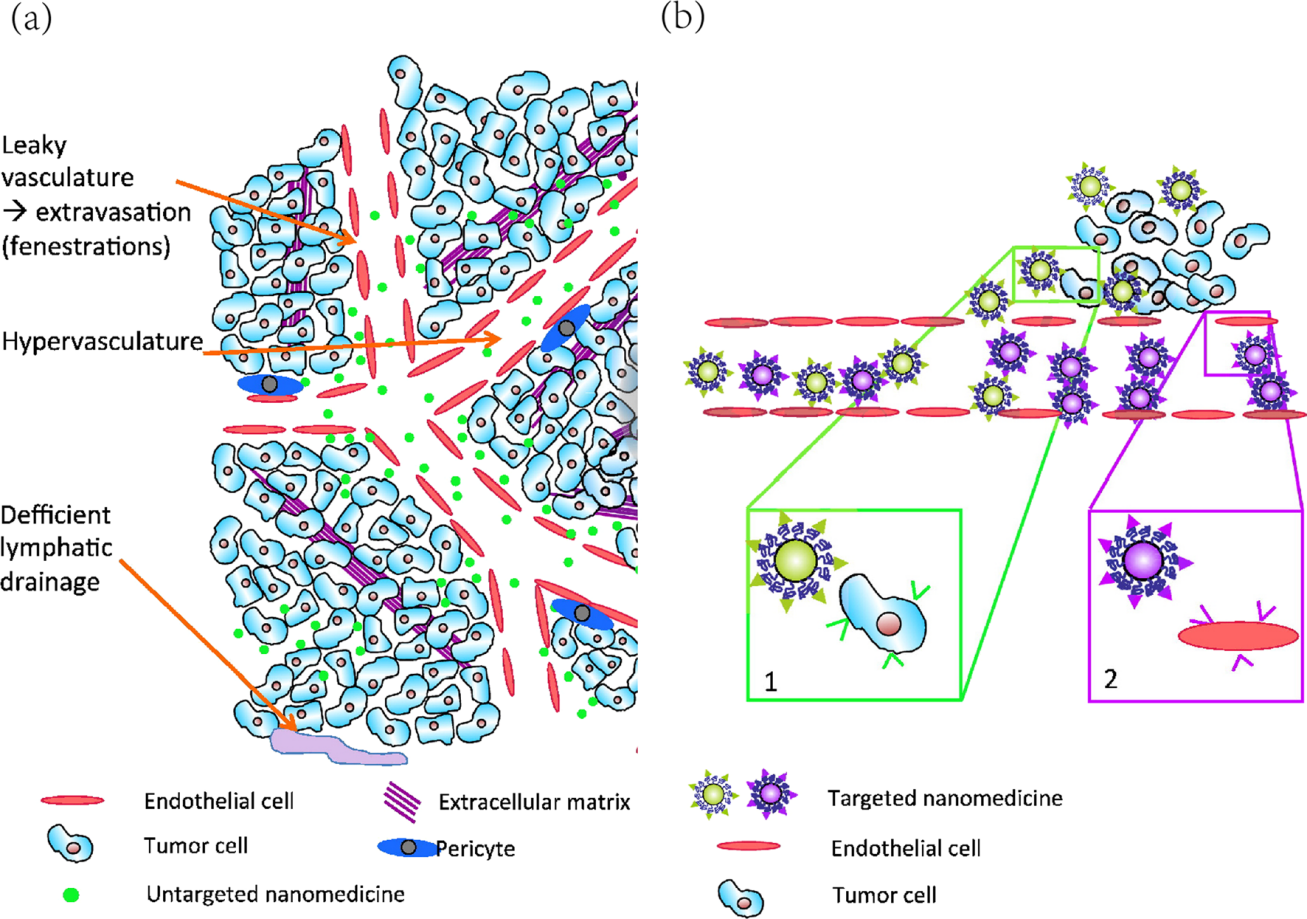
**a** Schematic representation of the conceptual passive targeting (EPR effect) of nanomedicine. **b** Active targeting of nanomedicine grafted with peptide or antibody able to bind specific receptors overexpressed by (1) cancer cells or (2) endothelial cells. Referred from [199] with permission

In 1986, Matsumura [193] proposed a phenomenon where nanoparticles with specific sizes displayed tumor accumulation properties. This tumoritropic performance was then recognized as enhanced permeability and retention (EPR) effect and played an indispensable role in nanoscale drug delivery therapy field (Fig. 9). With respect to tumorous tissues, the augmented angiogenesis is prone to accompany with wide inter-endothelial gap, poor vessel structural integrity and lymphatic reflux deficiency. These vascular alterations enable micromolecular substances (100 nm to 200 nm) to selectively permeate and retain in tumor surroundings [194]. The EPR effect endows drug delivery systems with passive targeting ability and contributes to increased therapeutic efficiency as well as reduced systemic side effects. However, controversy exists since the EPR effect tremendously varies intratumorally and intertumorally due to distinct tumor microenvironments and vascular densities [195, 196]. Warrenet al. [197] demonstrated that rare nanoparticles could passively penetrate into the tumor vessels of ovarian cancer, breast cancer and GBM. Based on mouse models, human tumorous tissues and mathematical models, conclusion was made that 97% of nanoparticles were transported through active process instead of passive diffusion [197]. Ding et al. [198] held opposite opinion that 87% renal cancer exhibited significant EPR effect, despite considerable heterogeneity was also observed in tumors with different size and gender (male samples showed intensified EPR) [198]. The EPR effect may eventually depend on the specific tumor pathophysiology, because differences were observed in the variety of tumor types. Although numerous EPR effect studies based on animal models achieved significant efficacy, the majority of which were failed to be translated into clinic [199]. Therefore, more intensive investigations should be conducted to fully illustrate the EPR effect behind nanoparticle drug delivery platforms.

Conventional anti-GBM drug delivery is invariably accompanied by unsatisfactory bio-distribution and systemic side effects. Since plenty of therapeutic agents against GBM cells fail to reach the action site and function in vivo, novel nanoscale biomaterials have been developed as vehicle to escort them. For one thing, nanoparticle delivery systems play an indispensable role in drug camouflage. The association between therapeutic agents and biomaterials can significantly improve the stability, solubility and bioavailability of drugs, which contributes to excellent pharmacokinetic performances (increased blood-drug concentration, prolonged half-life, etc.) of nanodrugs [2]. It is widely acknowledged that the most critical obstacle on GBM medication is BBB. Hydrophobic nanoparticles (liposomes, polymeric micelles) as well as modifications (transferrin/lactoferrin conjugation) were developed to increase the permeability of BBB. For another, actively GBM-specific property can be managed by nano-biomaterials. Several ligands such as FA, oligopeptide cRGD, Ang-2 and A7R [200] were associated with NPs to enhance their selectivity. The GBM-homing characteristic plays a pivotal role in decreased drug delivery frequency and reduced off-target effects.

The novel anti-GBM therapeutic agents range from chemotherapeutic drugs to chemokine, oligonucleotide, siRNA/shRNA, protein and etc. Therefore, diverse nanoparticle systems were utilized based on their unique characteristics and the associations with drugs. Sjoerd et al. [201] developed terminally functionalized PEG-*b*-PS and PEG-*b*-PDLLA libraries with 32 variants. The different amine groups on the terminal of PEG polymers allowed the chemical conjugations with specific drugs as well as the subsequent modifications through click chemistry reactions. Additionally, based on the physical characteristics such as size, charge, magnetism, hydrophobicity and photoexcitability, varied nanoparticles are selected to transport the drugs with diverse properties. Small interfering RNA (siRNA) is able to inhibit the expression of specific mRNA and is employed for cancer therapy. However, the vulnerable siRNA can be easily degraded by widely-existing RNase, repelled by cell membranes with the same negatively charge [202]. Cationic nanoparticles such as dendrimers, polymers and liposomes were used to carry siRNA via electrostatically association and protect it from rapid renal/phagocytic clearance [203].

Nanoscale DDS are also applied in diagnostic imaging, hyperthermia therapy and photodynamic therapy against GBM [204, 205]. Near-infrared (NIR) dye has been used as a noninvasive, real-time, in situ fluorescence imaging tool [206]. The cooperation of phtosensitizers and NPs could provide tumor targeting property [207], in which the active-targeting NIR exhibited profound potential on precise phototheranostics [9, 208]. In recent, more and more investigations focused on multimodal theranostic regimens. For example, superparamagnetic Fe_3_O_4_ nanoparticles was applied for magnetic fluid hyperthermia (MFH) and could be combined with chemotherapy drugs [209] as well as NIR hyperthermia [210]. Specifically, the administration routes of GBM delivery systems expanded to intravenous injection, nasal inhalation, and in situ implantation, which provided available strategies for appropriate treatment of GBM.

It was widely acknowledged that the significance of nanoscale biomaterials in drug delivery had been profoundly improved in the past few years due to their unique structures and properties. The prodigious progresses that have been made in biomedical field contributed to the diagnostic and therapeutic applications of anti-GBM nanoparticles. However, the majority of GBM nanoparticles investigations ended up at animal models and rare of these could enter preclinical stages. GBM is characterized by remarkably intra and inter-tumoral heterogeneity, therefore the inbred strain models lack complexity and diversity to reproduce human GBM. Except from BBB, biological or pathological barriers such as mononuclear phagocyte system, renal clearance, opsonization and blood tumor barrier (BTB) also have significant impacts on insufficient nanoparticles accumulation [211]. Additionally, limitations still exist on the safety issue. Although most nanoparticles exhibit excellent biocompatibility and biodegradability, fundamental investigations on the potential toxicity, immunogenicity, genotoxicity and bio-pharmacokinetics (absorption, distribution, metabolism, excretion) of each biomaterial must be scrupulously assessed in vivo. The bio-implants may trigger foreign body reactions (FBR) [212, 213], and hence the underlying influences of activated immune system on biomaterials, local homeostasis and even prognosis should be cautiously concerned. Despite the emergence of numerous nanoscale DDS for GBM treatment, explorations on effective dosage and bioavailability should also be scrupulously considered to minimize the side effects.

Currently, a great many of studies in drug delivery field still focused on pre-existing nanoparticles/biomaterials. The innovations of those DDS were mainly confined to superficial modifications and/or simply nanohybrid formation of multiple nanoparticles. Consequently, the development of novel biomaterials with superior vehicle capability is urgently needed for GBM therapy. Besides, despite most studies have described the material characterization of NPs thoroughly, investigations on the specific biological pathways behind DDS were comparatively inadequate.

In the future, more and more molecular targets behind GBM will be discovered. Therefore, the interdisciplinary nanoscale DDS that are able to precisely deliver the associated therapeutics will play a more significant role in GBM therapy.

## Conclusion

In this review, the major types of drug delivery systems in GBM were clarified, and each of them were systematically interpreted and exemplified. The advantages and limitations of drug delivery systems were discussed, mainly covering safety, design, synthesis, bio-distribution, functionalization and efficiency. In the discussion, challenges and opportunities for GBM nanodrug therapy were proposed.

## Data Availability

Not applicable.

## Abbreviations

GBM: Glioblastoma multiforme
NPs: Nanoparticles
DDS: Drug delivery system
BBB: Blood–brain-barrier
BTB: Blood tumor barrier
RME: Receptor-mediated endocytosis
AME: Absoptive-mediated endocytosis
CNTs: Carbon nanotubues
GBNs: Graphene-based nanoparticles
CQDs: Carbon quantum dots
MOFs: Metal–organic frameworks
LPs: Liposome particles
MSNs: Mesoporous slica nanoparticles
GNPs: Gold nanoparticles
PMs: Polymeric micelles
EPR: Enhanced permeability and retention
NIR: Near-infrared
DOX: Doxorubicin
TMZ: Temozolomide
CPT: Camptothecin
RAPA: Rapamycin
CXB: Celecoxib
PTX: Paclitaxel
MFH: Magnetic fluid hyperthermia
CMC: Critical micelle concentration
SPR: Surface plasmon resonance
